# Editorial: Interplay Between Autophagy and Metabolic Syndrome: Causes, Consequences and Therapeutic Challenges

**DOI:** 10.3389/fcell.2021.765778

**Published:** 2021-10-06

**Authors:** Stephen F. Rodrigues, Karen Y. Stokes, William T. Festuccia, Joilson O. Martins

**Affiliations:** ^1^Laboratory of Vascular Nanopharmacology, Department of Pharmacology, Institute of Biomedical Sciences, University of São Paulo (ICB/USP), São Paulo, Brazil; ^2^Department of Molecular and Cellular Physiology, Louisiana State University Health Shreveport, Shreveport, LA, United States; ^3^Department of Physiology and Biophysics, Institute of Biomedical Sciences, University of São Paulo (ICB/USP), São Paulo, Brazil; ^4^Laboratory of Immunoendocrinology, Department of Clinical and Toxicological Analyses, School of Pharmaceutical Sciences of University São Paulo (FCF/USP), São Paulo, Brazil

**Keywords:** autophagy, inflammation, cellular processes, metabolic syndrome, dyslipidemia

Metabolic syndrome (MetS) is a complex and heterogeneous cluster of interrelated metabolic and cardiovascular complications primarily composed of dyslipidemia, hypertension, impaired glucose homeostasis, visceral obesity, insulin resistance, non-alcoholic fatty liver disease, and chronic low-grade inflammation, among others (Ford et al., 2002; Alberti, 2009; Prieur et al., 2011; Maiano et al., 2016; Zmora et al., 2017). Although the prevalence of MetS has markedly increased in the last decades, the diverse molecular mechanisms promoting this cluster of conditions are still unknown. Recent pieces of evidence indicate that autophagy is directly implicated in MetS development. Autophagy is an intracellular process through which cell components are delivered to lysosomes for degradation. This process has several possible goals: to provide nutrients for energy production in periods of energy scarcity; to maintain cell homeostasis by degrading mal-functioning organelles and proteins; or to eliminate invading pathogens. With relevance to MetS, autophagy is dysregulated in adipocytes, hepatocytes, pancreatic β cells, endothelial cells, macrophages, among many other cell types, and has been associated with impairments in cell signaling, function and homeostasis in metabolic diseases such as visceral obesity, non-alcoholic fatty liver disease (NAFLD), type 2 diabetes, and atherosclerosis (Lee et al., 2019). Therefore, understanding the molecular mechanisms by which autophagy is implicated in MetS may open the possibility for the development of novel therapeutic strategies to prevent and treat this cluster of metabolic diseases.

In this Research Topic, we gathered several Review and Original Research articles that shed light on the mechanisms through which autophagy is regulated and implicated in the development of MetS. Among those, two exceptional review articles address the intricate interrelationship between autophagy, nutrient availability, and growth factor signaling in the insulin-target tissues skeletal muscle, liver, and white adipose tissue. In a very interesting mini-review, Deleyto-Seldas and Efeyan discuss how targeting of rapamycin (mTOR) complex 1 (mTORC1) and complex 2 (mTORC2) inhibits autophagy in response to nutrients and growth factors through a mechanism that involves the acute blockade of autophagosome formation, as well as a reduction in the transcription of several components of autophagy machinery via inhibition of TFEB and FoxO1. In an elegant and insightful article, Frendo-Cumbo et al. review the impact of obesogenic and diabetogenic conditions upon autophagy in insulin-target tissues with an emphasis in the implication of autophagy deregulation in the development of insulin resistance. Mechanistically, this seems to directly involve mTORC1-ULK and IRS1 and indirectly, endoplasmic reticulum stress, inflammation and lipotoxicity.

Two interesting review articles have addressed the involvement of autophagy in metabolic complications affecting the liver. Indeed, Ramos et al. reviewed all articles published in the last 10 years that evaluated liver autophagy in rodent models of nutritional, genetic, and pharmacologic NAFLD and obesity. These articles were discussed regarding of how steatosis affects autophagy at the molecular level and how can pharmacological interventions and autophagy knockout models help to elucidate the interplay between autophagic flux and steatosis. Álvarez-Mercado et al., on the other hand, discussed the implication of autophagy in the ischemia-reperfusion injury inherent to surgical liver resection or transplantation in patients with MetS. Important modulators of autophagy in these conditions namely endoplasmic reticulum and oxidative stresses, the mitogen-associated protein kinase (MAPK), along with the energy sensor AMP-activated protein kinase (AMPK), were deeply discussed.

In a mini-review article, Frisardi et al. emphasized the potential multifunctional role of high mobility group box 1 (HMGB1) protein as a promising biomarker candidate and therapeutic target for the early stage of MetS. HMGB1 is a highly conserved protein that, in addition to its nuclear function of maintaining genomic structure, is also secreted, either passively from damaged cells, or actively from activated immune cells, under stress. Secreted HMGB1 primarily acts as a danger associated molecular pattern (DAMP) that activates RAGE and TLR4 receptors. Several studies have shown that HMGB1 is a critical regulator of autophagy. Interestingly, there is evidence that glucagon-like peptide-1 receptor (GLP-1R) activation can inhibit HMGB1, and a role for the GLP-1R in the autophagic flux was identified by Fang et al. in an original article in which they used liraglutide, a GLP-1RA agonist, and experimental models of NAFLD. They showed that liraglutide attenuated hepatic steatosis via activation of autophagic flux via the GLP-1R-TFEB-mediated autophagy-lysosomal pathway.

The inflammasome, a complex composed of many proteins, is an important part of the innate immune system and is used to detect the presence of infection, pathogens, and metabolic alarms in cells. In a review article, Lv et al. discussed two mechanisms by which autophagy and the NLRP3 inflammasome interact and contribute to metabolic disorders progression. The first one concerns the inhibition of the NLRP3 inflammasome due to a decrease in reactive oxygen species (ROS) production caused by autophagy-mediated scavenging of damaged mitochondria. Mitochondrial ROS promote the transcription of NLRP3 and pro-IL-1β, via NFκB activation. The second discussed mechanism concerns the role of autophagy as a system that directly degrades components of the inflammasome complex. Two other papers also invoked a role for oxidative stress in the deregulated autophagy of MetS. Nwose and Bwititi discussed the role of erythrocyte oxidative stress (EOS) in autophagy, especially in diabetes, and the use of clinical laboratory tests to measure oxidative stress as an indirect method to assess autophagy in MetS. They also highlighted that abnormal cholesterol can exacerbate oxidative stress to increase autophagy in diabetes. Moreover, Su et al. discussed the mechanism of mitochondrial dysfunction in metabolic disorders and the potential of targeting mitophagy with phytochemicals for the treatment of metabolic disorders.

Endothelial dysfunction plays a central role in linking MetS with neurological disorders. Maiuolo et al. discussed the fundamental role of autophagy in the maintenance of endothelial cell homeostasis and the implication of autophagy deregulation in the onset of MetS. Moreover, a positive correlation has been found between MetS and retinopathy, primarily at the microvascular level, in patients without a history of diabetes. Paz et al. investigated beyond the vascular component by analyzing functional changes as well as neuronal and glial responses in retinas of Apolipoprotein E knockout (ApoE-KO) mice fed with 10% w/v fructose diet. This is relevant because neuronal autophagy is important for the maintenance of neuronal homeostasis and vitality as neurons are particularly vulnerable to altered and/or impaired autophagy. In fact, autophagy has been found to be of particular importance in the attenuation of neurological disorders associated with MetS. Paz et al. demonstrated that ApoE-KO mice fed for 2 months with a fructose enriched diet presented with biochemical alterations representative of MetS. This was accompanied by elevated blood retinal barrier permeability and impaired retinal function that was associated with decreased astrocyte integrity and muted glial activation. While these changes occurred in the absence of notable alterations in microvascular density or angiogenesis, there was an associated impairment of intracellular recycling mechanisms.

Pant et al. provide an updated review of the interplay between adipose tissue function, adipokines, systemic inflammatory profile and metabolic health, including, the transcriptional and epigenetic regulation of adipogenesis by environment factors, which are responsible for the transgenerational inheritance of the disease. In addition, atherosclerosis is characterized by lipid accumulation and increased inflammatory cytokines in the vascular wall. This is precipitated by endothelial cell and vascular smooth muscle cell dysfunction, and results in foam cell formation from monocytes/macrophages. Xu et al. highlighted the role of autophagy and MetS characteristics in the pathogenesis of atherosclerosis and potential therapeutic drugs that target these molecular mechanisms.

Finally, in an insightful review article, Ro et al. discussed recent studies that investigated Sestrins, an emerging dynamic group of proteins that act as nutrient, stress, and redox state sensors in cells, and may serve as potential preventive or therapeutic targets to counteract metabolic diseases including MetS and aging.

In conclusion, this Research Topic gathered 14 review and original articles addressing novel and updated aspects related to autophagy and MetS, covering molecular mechanisms and cellular processes in both preclinical and clinical models of study, and revealing potential treatments.

## Author Contributions

All authors contributed equally to the editorial article and approved the submitted version.

## Funding

This work was supported by grants from Fundação de Amparo à Pesquisa do Estado de São Paulo (FAPESP) to SR (#2014/05146-6, 2020/07212-7), JM (#2020/03175-0), WF (#2015/19530-5, 2019/01763-4 and 2020/04159-8) and Conselho Nacional de Desenvolvimento Científico e Tecnológico (CNPq) to JM (#310993/2020-2) and WF (#301756/2019-8), and the National Heart, Lung, and Blood Institute of the NIH to KS (R01HL134959).

## Conflict of Interest

The authors declare that the research was conducted in the absence of any commercial or financial relationships that could be construed as a potential conflict of interest.

## Publisher's Note

All claims expressed in this article are solely those of the authors and do not necessarily represent those of their affiliated organizations, or those of the publisher, the editors and the reviewers. Any product that may be evaluated in this article, or claim that may be made by its manufacturer, is not guaranteed or endorsed by the publisher.
